# Stem cell-derived extracellular vesicles for high-altitude skin injuries: pathological mechanisms, advanced Nano-delivery, and translational perspectives

**DOI:** 10.3389/fbioe.2026.1847706

**Published:** 2026-07-08

**Authors:** Yilin Wang, Huifang Yang, Kexin Tang, Tang Tang, Jing Guo

**Affiliations:** 1 School of Clinical Medicine, Chengdu University of Traditional Chinese Medicine, Chengdu, Sichuan, China; 2 Department of Dermatology, Affiliated Hospital of Chengdu University of Traditional Chinese Medicine, Chengdu, Sichuan, China

**Keywords:** clinical translation, high-altitude skin injury, nanoparticle delivery systems, stem cell-derived extracellular vesicles, stimuli-responsive hydrogels

## Abstract

The extreme high-altitude environment—characterized by hypobaric hypoxia, intense ultraviolet radiation, and severe cold and aridity—imposes complex physical and biochemical stresses that synergistically drive pronounced cutaneous oxidative stress and barrier collapse. Given the limited capacity of conventional interventions to restore damaged cutaneous microenvironments, stem cell-derived extracellular vesicles (SC-EVs) have emerged as a promising cell-free regenerative modality for multitarget intervention. By delivering a diverse repertoire of bioactive molecules, SC-EVs concurrently help restore reactive oxygen species (ROS) homeostasis, attenuate inflammatory cascades, modulate angiogenesis, and mitigate extracellular matrix (ECM) degradation, thereby promoting coordinated restoration of cutaneous homeostasis. Nevertheless, the rapid *in vivo* clearance and limited structural stability of native EVs severely bottleneck their clinical translation. To surmount these limitations, advanced nanodelivery platforms—such as stimuli-responsive hydrogels and liposomal hybrids—may provide strategies to improve the targeted spatial retention, spatiotemporal release, and overall bioavailability of EVs. This review systematically summarizes and discusses the molecular mechanisms by which SC-EVs counteract composite high-altitude skin injuries. By bridging cutting-edge nanodelivery technologies with altitude-specific therapeutic adaptations, we delineate a potential translational framework for developing non-invasive nanotherapeutics tailored for the precise prevention and treatment of high-altitude dermatological damage.

## 1 Introduction

The fundamental challenge to cutaneous health at high altitudes lies in the synergistic impact of extreme environmental stressors—including hypobaric hypoxia, intense ultraviolet (UV) radiation, and cold-arid conditions. These factors synergistically induce oxidative stress and chronic inflammation, culminating in complex clinical phenotypes such as accelerated photoaging, impaired barrier function, and refractory wounds (Singh, 2017; Zhang et al., 2021a; Thareja et al., 2025; Chatterjee and Hemdani, 2025; Singh et al., 2013; Lin et al., 2025). While indigenous populations have evolved partial genetic adaptations (e.g., variants in EPAS1 and GNPAT) to optimize hypoxic responses and UV protection, these mechanisms offer no reprieve for the vast number of short-term residents or individuals with compromised regenerative capacity (Bai et al., 2022; Yang et al., 2022). Current clinical interventions rely heavily on physical sunscreens and exogenous antioxidants; however, these modalities are inherently circumscribed by their superficial action, poor bioavailability, and suboptimal targeting (Schrempf et al., 2016; Al-Niaimi and Chiang, 2017; Amri et al., 2012). Furthermore, the logistical complexities and erratic weather patterns characteristic of high-altitude regions exacerbate the difficulty of resource delivery, rendering conventional strategies insufficient for achieving repair at the cellular level (Gairolla et al., 2025; Perez et al., 2025).

In recent years, SC-EVs have emerged as a promising cell-free therapeutic modality to address these multifaceted challenges. A growing body of preclinical evidence demonstrates that SC-EVs, by delivering a diverse cargo of bioactive molecules, can antagonize hypoxia- and UV-induced skin damage across multiple biological fronts—including the activation of the Nrf2/GPX4 axis to restore redox homeostasis, suppression of inflammatory cascades, and promotion of collagen synthesis and angiogenesis (Huynh et al., 2025; Han et al., 2024a; Yan et al., 2023a; Gui et al., 2025). Although EVs derived from distinct tissues (e.g., adipose, umbilical cord, or bone marrow) exhibit varied molecular profiles (Huynh et al., 2025; Yan et al., 2023a; Gui et al., 2025; Zha et al., 2023; Liu et al., 2024), their core functions remain complementary. This functional diversity provides a versatile mechanistic “toolbox” tailored to the pathological complexity of high-altitude composite injuries.

Despite this promise, translating these laboratory-scale advancements into clinically viable products for high-altitude applications remains hindered by two critical “knowledge gaps.” First, from a biological efficacy perspective, native EVs suffer from intrinsic limitations such as rapid *in vivo* clearance and inadequate targeting, which significantly curtail their bioavailability and therapeutic impact (Palakurthi et al., 2024). Second, from an engineering and translational standpoint, the extreme high-altitude environment—marked by drastic temperature fluctuations and mechanical stress during long-distance transport—imposes rigorous demands on the stability and storage of EVs formulations. To date, validated, integrated solutions for these specific scenarios remain elusive. While preliminary studies have explored the use of delivery systems, such as hydrogels and lipid nanoparticles, to enhance sEV retention (Han et al., 2024b; Park et al., 2022), and investigated lyophilization or smart logistics (e.g., drones) to improve transport (Oakey et al., 2021; Sivanantham and Jin, 2022; Vakil et al., 2023; Aggarwal et al., 2024; Abduljabbar et al., 2019), these technological modules remain fragmented. They have yet to undergo systematic integration or adaptive validation within the specialized context of high-altitude skin repair.

Consequently, this review aims to systematically synthesize existing evidence to dissect the aforementioned translational bottlenecks. By exploring the feasibility of integrating advanced delivery technologies with altitude-specific adaptation strategies, we seek to provide translational roadmap for the development of EVs-based therapeutics optimized for precision skin protection and repair in high-altitude environments.

## 2 The multidimensional mechanistic network of high-altitude environment-induced skin injury

**FIGURE 1:**
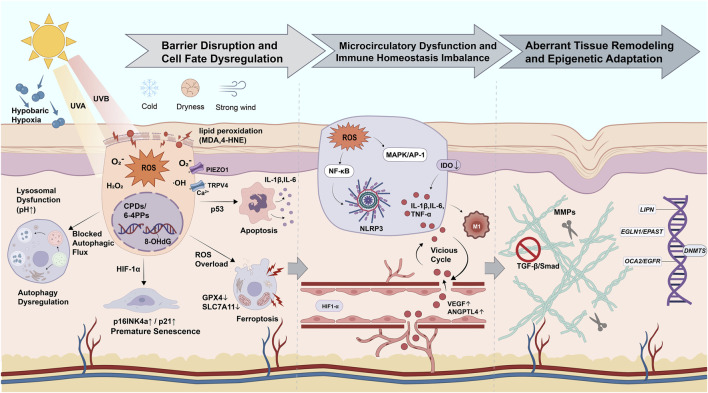
The Cascading Pathophysiological Mechanism Network of Skin Injury Induced by Extreme High-Altitude Environments. (All panels are original illustrations created by the authors using Adobe Illustrator).

Current *in vivo* models directly investigating high-altitude skin injury remain limited, and most available evidence is derived from studies of ultraviolet (UV)-induced damage, hypoxic stress, chronic wounds, or normoxic ischemic models. However, skin injury under high-altitude conditions is unlikely to represent a simple additive effect of individual stressors. Rather, it constitutes a complex pathological process driven by the combined actions of hypoxia, excessive UV radiation, low temperature, strong wind, and reduced atmospheric pressure. Based on current advances in high-altitude medicine, cutaneous biology, and tissue repair research, this section systematically integrates the potential pathological cascade underlying high-altitude skin injury from three interconnected dimensions: barrier disruption, microcirculatory and immune dysregulation, and aberrant tissue remodeling. Through this framework, we aim to provide a mechanistic basis for the subsequent development of SC-EV-based therapeutic strategies (Figure 1).

### 2.1 Barrier disruption and cell fate dysregulation driven by physical and biochemical stress

The initial cutaneous injury induced by high-altitude environments fundamentally arises from the persistent disruption of epidermal barrier homeostasis under multidimensional environmental stress. Excessive UV radiation, hypoxia, low temperature, and strong wind collectively constitute a complex network of biochemical and physical insults, driving the progression from barrier dysfunction to deep cellular injury and impaired tissue repair.

At the biochemical level, oxidative stress synergistically induced by UV radiation and hypoxia is considered a pivotal early event in high-altitude skin injury. UV exposure directly penetrates the epidermis and stimulates keratinocytes and melanocytes to generate excessive ROS, including superoxide anions and hydroxyl radicals, thereby triggering severe lipid peroxidation characterized by the accumulation of malondialdehyde (MDA) and 4-hydroxynonenal (4-HNE) (de Jager et al., 2017; Mucha et al., 2025; Wei et al., 2024). In particular, UVB radiation directly induces the formation of cyclobutane pyrimidine dimers (CPDs), resulting in DNA damage and genomic instability (Lee et al., 2020). Concurrently, hypoxic stress stabilizes hypoxia-inducible factor-1α (HIF-1α) and activates NADPH oxidase (NOX)-related pathways, further amplifying intracellular ROS production (Tafani et al., 2016; Tian et al., 2022; Feng et al., 2023; Nisar et al., 2025). Field studies have similarly demonstrated elevated lipid peroxidation levels, including increased MDA accumulation, following high-altitude exposure, accompanied by reduced activities of antioxidant defense enzymes such as superoxide dismutase (SOD) and glutathione peroxidase (GPX) (Dosek et al., 2007; Siques et al., 2018; Bakonyi and Radak, 2004). Collectively, these findings indicate the presence of sustained redox imbalance under high-altitude conditions.

In addition to oxidative stress, physical environmental alterations may further contribute to epidermal barrier dysfunction. Strong wind, low temperature, and hypoxia-associated tissue edema under high-altitude conditions may alter the local mechanical microenvironment and thereby disrupt epidermal homeostasis. Previous studies have shown that keratinocytes are capable of sensing abnormal mechanical stimuli through mechanosensitive ion channels such as PIEZO1 and TRPV4, which mediate Ca^2+^ influx and downstream mechanotransductive responses (Mikesell et al., 2022; Moehring et al., 2018). Persistent abnormal mechanical tension may further disturb keratin homeostasis and keratinocyte differentiation programs. Among these alterations, aberrant expression of the stress-associated keratin Krt16 is frequently linked to defective epidermal repair, whereas structural keratins such as Krt9 may also exhibit dysregulated expression under chronic stress conditions (Fu et al., 2014; Zieman et al., 2019). Moreover, hypoxia has been shown to impair filaggrin expression and interfere with stratum corneum maturation (Park et al., 2016). These alterations may collectively compromise epidermal barrier integrity and aggravate skin injury under the compounded stresses of high-altitude environments. Nevertheless, direct evidence linking mechanotransduction abnormalities to high-altitude skin pathology remains limited, and the precise mechanisms involved require further investigation.

Persistent barrier disruption and oxidative stress subsequently induce profound disturbances in cellular fate regulation, which may represent a critical driver of impaired wound repair. Emerging evidence suggests that abnormal mechanical stimulation can activate mechanosensitive transcriptional programs involving YAP/TEAD signaling, thereby affecting keratinocyte differentiation and regenerative capacity (Biggs et al., 2020). Simultaneously, excessive ROS accumulation may suppress glutathione peroxidase 4 (GPX4) activity, leading to lipid peroxide accumulation and the initiation of ferroptosis (Chen et al., 2025; Rezzani et al., 2024; Zhou et al., 2024). In parallel, UVB-induced stabilization of p53 promotes keratinocyte apoptosis (Rezzani et al., 2024; Zhou et al., 2024; Yu et al., 2024; Carvalho et al., 2024), whereas UVA exposure and chronic hypoxia may accelerate cellular senescence and the accumulation of damaged biomolecules through autophagy impairment and upregulation of senescence-associated factors such as p16INK4a (Nisar et al., 2025; Ma et al., 2022; Zhang et al., 2025a; Otero-Albiol and Carnero, 2021). Sustained activation of these interconnected stress and cell death pathways may ultimately impair early re-epithelialization and contribute to the development of chronic skin injury under high-altitude conditions.

### 2.2 Microcirculatory dysfunction and immune homeostasis imbalance

During the intermediate stage of pathological progression, upstream oxidative stress and tissue injury signals further evolve into persistent microcirculatory dysfunction and immune imbalance. As a central pathological mediator in the high-altitude composite environment, ROS predominantly activate two major inflammatory pathways: nuclear factor-κB (NF-κB) and mitogen-activated protein kinase/activator protein-1 (MAPK/AP-1). Activation of NF-κB promotes the assembly of the NLRP3 inflammasome and induces the expression of pro-inflammatory cytokines, including interleukin-1β (IL-1β), interleukin-6 (IL-6), and tumor necrosis factor-α (TNF-α). In parallel, NF-κB signaling also upregulates cyclooxygenase-2 (COX-2), thereby enhancing prostaglandin synthesis and amplifying local inflammatory responses (Salminen et al., 2022; Hasegawa et al., 2016; Gromkowska-Kępka et al., 2021). Meanwhile, activation of the MAPK/AP-1 pathway further increases the expression of matrix metalloproteinases (MMPs) and pro-inflammatory mediators, while interacting with apoptosis-related signaling pathways to exacerbate inflammatory injury (He et al., 2022; Nys et al., 2012; Yan et al., 2023b).

Compared to the rapid hyperemic response in normobaric mechanical wounds, high-altitude cutaneous microvasculature exhibits profound endothelial maladaptation. Clinical hemodynamic data reveal that under acute hypoxic exposure equivalent to 4,500 m, basal microvascular blood flow exhibits a compensatory surge; however, the amplitude of reactive hyperemia in response to local ischemia declines sharply (from a normoxic baseline of approximately 58%–45%), confirming that the endothelial vasodilatory reserve is markedly impaired (Treml et al., 2018). Crucially, despite compensatory increases in basal perfusion, oxygen delivery efficiency remains substantially compromised due to endothelial dysfunction, vascular leakage, and microthrombotic obstruction. Furthermore, under the dual stress of hypoxia and aberrant mechanical forces (e.g., elevated interstitial pressure secondary to edema and sustained external compression), sustained HIF-1 activation has been associated with increased plasminogen activator inhibitor-1 (PAI-1) expression and a prothrombotic microvascular phenotype (Kaneko et al., 2015).

Regarding vascular remodeling, hypoxia drives the overexpression of vascular endothelial growth factor (VEGF) and Angiopoietin-like 4 (ANGPTL4), yielding structurally malformed and hyperpermeable immature capillary beds (Hu et al., 2016; Hartono et al., 2022). The subsequent interplay between edema and deep tissue ischemia further dysregulates HIF-2α expression (Gottrup et al., 2017; Chen et al., 2022) and promotes its crosstalk with the NF-κB pathway. This dynamic establishes a self-sustaining vascular-inflammatory amplification circuit—where vascular leakage exacerbates inflammatory infiltration, and inflammatory factors reciprocally disrupt the endothelial barrier (D'Ignazio et al., 2016; Pham et al., 2021; Cho et al., 2012)—ultimately driving the local microenvironment into a state of adaptive failure.

Synchronized with this vascular destabilization is the arrested polarization of the local immune system. Under the combined stress of deep tissue hypoxia and inflammatory infiltration, indoleamine 2,3-dioxygenase (IDO)—a key innate immune regulatory enzyme—is significantly downregulated (Wang et al., 2014; Schmidt et al., 2013). This suppresses the anti-inflammatory cytokine IL-10 while sustaining TNF-α expression (Sindrilaru et al., 2011; François et al., 2012; Wang et al., 2006), thereby skewing macrophage polarization toward a persistent M1-dominant inflammatory state. Moreover, the hypoxic microenvironment epigenetically restricts histone demethylase activity, directly downregulating the transcription of essential chemokine signaling molecules, including receptors (e.g., CCR1, CCR5) and their corresponding ligands (e.g., CCL2) (Tausendschön et al., 2011). Consequently, local immune cell recruitment and turnover are impaired, substantially compromising the early scavenger function essential for subsequent tissue remodeling (Wang and Shi, 2020).

### 2.3 Aberrant tissue remodeling and epigenetic adaptation

Downstream mechanisms center on the critical disruption of ECM homeostasis and the epigenetic consolidation of damage phenotypes. UV radiation significantly upregulates the expression of MMP-1, MMP-3, and MMP-9, precipitating the excessive degradation of type I and III collagens alongside elastin. This proteolytic degradation is the direct proximate cause of photoaging phenotypes, such as skin wrinkling and laxity (Quan et al., 2009; Pittayapruek et al., 2016; Fisher et al., 1997; Hachiya et al., 2009; Zorina et al., 2022). This process is intimately associated with the suppression of the transforming growth factor-β (TGF-β)/Smad signaling pathway, which diminishes *de novo* collagen synthesis and completely dismantles the equilibrium between ECM synthesis and degradation (Quan et al., 2004; Quan et al., 2001; Varani et al., 2002). Hypoxia, conversely, drives aberrant collagen modifications and exacerbates defective ECM remodeling by modulating collagen cross-linking-associated genes (e.g., PLOD2, LOXL2) via the HIF-1α axis (Brereton et al., 2022).

Epigenetic modifications, particularly DNA methylation, serve as the pivotal mechanisms by which damage signals are chronically sustained and molecularly “remembered.” Hypoxic environments modulate DNA methyltransferase (DNMT) activity via the HIF pathway, thereby remodeling the methylation landscape of hypoxia-responsive genes (e.g., EGLN1, EPAS1) to dictate organismal adaptation (Zhang et al., 2025b; Mata-Greenwood et al., 2017). UV-induced oxidative stress similarly perturbs established methylation patterns, exemplifying this by silencing tumor suppressor genes like p16INK4a (Katiyar et al., 2012). Population-based studies have corroborated that high-altitude exposure elicits persistent DNA methylation alterations in genes intricately associated with barrier function (LIPN), hypoxic response (RBX1, PSMA8), and pigmentation (OCA2, EGFR). These entrenched “epigenetic memories” likely constitute a profound molecular basis for the distinctive phenotypic signatures of high-altitude skin (Zhang et al., 2024a; Pryor et al., 2025). Collectively, dysregulated tissue remodeling and epigenetic modifications constitute the terminal nodes of the pathogenic network, providing a robust theoretical rationale for the development of targeted, precision interventions.

## 3 The role of stem cell-derived extracellular vesicles and biomimetic derivatives in high-altitude skin repair

### 3.1 The role of mesenchymal stem cell-derived extracellular vesicles

**FIGURE 2:**
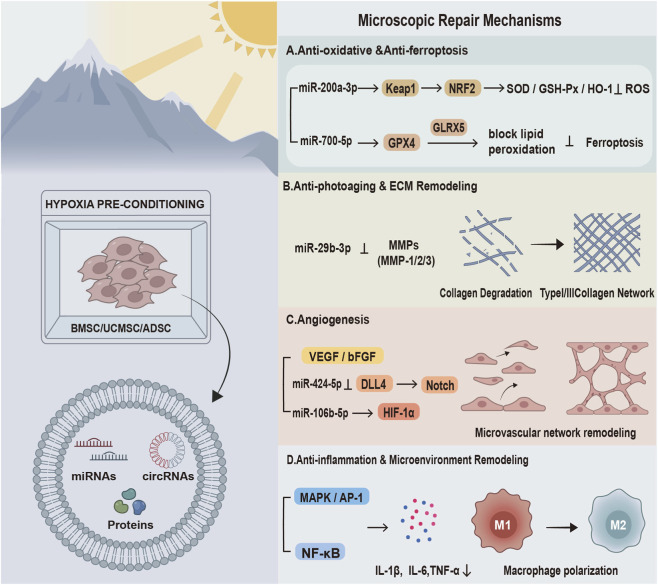
Multidimensional repair mechanisms of stem cell‐derived extracellular vesicles and their derivatives in antagonizing skin injury. **(A)** Anti‐oxidative and anti‐ferroptosis effects, involving the regulation of ROS‐related pathways and inhibition of lipid peroxidation and ferroptosis. **(B)** Anti‐photoaging and extracellular matrix (ECM) remodeling, involving the suppression of MMP‐mediated collagen degradation and restoration of the type I/III collagen network. **(C)** Angiogenesis, involving VEGF/bFGF‐related signaling and regulation of DLL4/Notch and HIF‐1α pathways to promote microvascular network remodeling. **(D)** Anti‐inflammation and microenvironment remodeling, involving modulation of MAPK/AP‐1 and NF-κB-related inflammatory responses and macrophage polarization. (All panels are original illustrations created by the authors using Adobe Illustrator).

SC-EVs have emerged as bioactive nanovesicular mediators for cell-free regenerative therapy. By delivering bioactive molecules—including miRNAs and proteins—they coordinately modulate critical pathological processes such as oxidative stress, inflammation, angiogenesis, and ECM remodeling (Table 1; Figure 2). Their biological composition and targeted repair efficacy are dependent on the tissue-specific origin of the parent cells, donor status, culture microenvironment, and isolation protocols. This inherent preparation heterogeneity dictates the distinct therapeutic focus of EVs derived from various sources during tissue regeneration.

**TABLE 1:** Therapeutic effects and mechanisms of EVs from various stem cell sources on high-altitude skin injury.

Extracellular vesicle sources	Core delivered molecules	Key signaling pathways/Targets	Core repair mechanisms and biological effects	References
Bone Marrow Mesenchymal Stem Cell-Derived Extracellular Vesicles (BMSC-EVs)	miR-29b-3p, miR-424–5p, miR-106b-5p, VEGF	MMP-2/1/3, MAPK/AP-1 signaling pathway, DLL4/Notch axis, HIF-1α	Blocks type I collagen degradation and promotes its synthesis; attenuates oxidative stress (decreases MDA, increases SOD/GSH-Px); promotes endothelial cell tube formation and microvascular remodeling	Yan et al. (2023a), Yan et al. (2023b), Yuan et al. (2021), Xiong et al. (2024), Cao et al. (2025)
Umbilical Cord Mesenchymal Stem Cell-Derived Extracellular Vesicles (UCMSC-EVs)	miR-200a-3p, miR-125 b	Keap1-Nrf2, NF-κB pathway, TP53INP1	Reduces ROS and DNA damage (8-OHdG) and repairs mitochondria; downregulates pro-inflammatory cytokines (IL-1β, IL-6); attenuates endothelial apoptosis and accelerates wound healing	Gui et al. (2025), Wang et al. (2020a), Liu et al. (2021), Zhang et al. (2021b), Angelina et al. (2025), Zhang et al. (2015)
Adipose-Derived Stem Cell Extracellular Vesicles (ADSC-EVs)	Circular RNA circ-Ash1L, GLRX5	circ-Ash1L/miR-700–5p/GPX4, ERK/MAPK pathway	Scavenges ROS, inhibits lipid peroxidation and ferroptosis; suppresses apoptosis/pyroptosis, promotes macrophage polarization towards the anti-inflammatory M2 phenotype; increases collagen density and delays photoaging	Huynh et al. (2025), Zha et al. (2023), Liu et al. (2024), Xiong et al. (2020), Zhang et al. (2025c), Wang et al. (2017)
Induced Pluripotent Stem Cell-Derived Extracellular Vesicles (iPSC-EVs)	miR-16–5p, miR-762	p38/MAPK, integrin β1, and other signaling pathways	Promotes the proliferation and migration of fibroblasts and keratinocytes; upregulates type I collagen and inhibits MMPs to regulate senescence; accelerates angiogenesis and wound healing	Oh et al. (2018), Liang et al. (2025), Meng and Guo (2023), Kobayashi et al. (2018), Yan et al. (2020), Bo et al. (2022)
Epidermal Stem Cell-Derived Extracellular Vesicles (EpSC-EVs)	miR-200b-3p, miR-203a-3p	miR-200b-3p/SYDE1/RAS/ERK, PI3K/AKT/mTOR, PKN1-cyclin	Attenuates endothelial damage and inhibits excessive autophagy; promotes skin appendage and vascular regeneration; exhibits anti-fibrotic, anti-inflammatory, and multiple pro-healing effects	Zhang et al. (2020), Xu et al. (2024), Zhao et al. (2025), Kang et al. (2024)
Hair Follicle Stem Cell-Derived Extracellular Vesicles (HFSC-EVs)	miR-125b-5p, lncRNA H19	TGF-β1/Smad, lncRNA H19/NLRP3	Enhances collagen synthesis (COL-1/3) to repair wrinkles; inhibits pyroptosis to promote repair	Cui et al. (2024), Yang et al. (2023)
Endothelial Progenitor Cell-Derived Extracellular Vesicles (EPC-EVs)	Rich in VEGF, FGF-2, and other factors	RAF/ERK, MAPK/NF-κB, AGE-RAGE pathways	Promotes angiogenesis and ameliorates angiogenic dysfunction; mitigates oxidative stress injury; promotes collagen maturation and reduces scarring	Ellistasari et al. (2022), Zhang et al. (2016), Jia et al. (2019), Wu et al. (2018), Xu et al. (2020), Oh et al. (2021)

Although *in vivo* studies directly targeting high-altitude skin injury are currently lacking, emerging evidence indicates that SC-EVs effectively counteract the core pathogenic factors of high-altitude environments. Viewed through the lens of intercellular communication, a conserved bidirectional molecular crosstalk exists between the hypoxic response and EV biology. On the one hand, microenvironmental hypoxic stress induces the adaptive remodeling of the parent MSC transcriptomic landscape and cargo packaging hierarchy. This regulates EV biogenesis and secretion, driving the targeted sorting of specific bioactive non-coding RNAs (e.g., miRNAs, circRNAs) and functional proteins (Bister et al., 2020). On the other hand, these hypoxia-adapted EVs subsequently reprogram recipient-cell signaling networks, thereby shifting pathological hypoxic responses toward adaptive regenerative phenotypes (Bister et al., 2020). This dynamic intercellular signaling ultimately orchestrates local networks to promote angiogenic remodeling, block mitochondrial apoptotic cascades, and suppress ferroptotic signaling associated with lipid peroxidation overload.

#### 3.1.1 Bone marrow mesenchymal stem cell-derived extracellular vesicles (BMSC-EVs)

Regarding UV radiation-induced injury, cumulative evidence substantiates the reparative efficacy of BMSC-EVs. For instance, in UVB-induced skin photoaging models, BMSC-EVs enriched in miR-29b-3p suppress MMP-2 signaling, subsequently downregulating MMP-1 and MMP-3 expression. This intervention impedes type I collagen degradation, thereby mitigating collagen degradation and partially restoring collagen homeostasis (Yan et al., 2023a). Concurrently, this regulatory axis attenuates oxidative stress (reducing MDA levels while elevating SOD and GSH-Px activities) and promotes fibroblast migration while suppressing apoptosis in a dose-dependent manner (optimal concentration: 25 μg/mL) (Yan et al., 2023a). Furthermore, a core mechanism of BMSC-EVs involves dampening the UVB-activated MAPK/AP-1 signaling pathway by downregulating p-p38, c-Jun, and c-Fos—an effect that is reversible by p38 agonists (Yan et al., 2023b).

The pathological progression of high-altitude skin injury is consistently accompanied by a sustained hypoxic microenvironment. While this feature constitutes a primary barrier to wound repair, it also provides a targeted strategy for optimizing BMSC-EV function. Hypoxic preconditioning (typically 0.5%–2% O_2_) not only consistently enhances EV secretion efficiency without altering their fundamental characteristics but also induces a profound reconfiguration of their cargo. Studies demonstrate that under hypoxic signaling, parent BMSCs undergo specific cargo sorting: their EVs actively enrich higher levels of pro-angiogenic macromolecular proteins, such as VEGF (Yuan et al., 2021). Simultaneously, differential regulation occurs at the non-coding RNA level, evidenced by the upregulation of miR-424–5p (Xiong et al., 2024) and the specific downregulation of miR-106b-5p (Cao et al., 2025), generating distinct hypoxia-adapted EVs.

Upon internalization by endothelial cells, these hypoxia-adapted BMSC-EVs profoundly remodel the wound microenvironment. Mechanistically, EV-enriched miR-424–5p targets the 3′-UTR of DLL4 mRNA, blocking its translation and dampening the subsequent pathological activation of the downstream Notch (Notch1/Notch2) pathway (Xiong et al., 2024). This post-transcriptional silencing mechanism relieves endogenous inhibitory constraints on endothelial sprouting and synergistically upregulates key target genes such as Ang1 and Flk1 (Xiong et al., 2024), thereby driving early microvascular network remodeling. Meanwhile, the specific downregulation of miR-106b-5p within the EV cargo constitutes a complementary regulatory axis. Upon entering recipient cells, this relieves the post-transcriptional repression of endogenous hypoxia-inducible factor-1α (HIF-1α), restoring HIF-1α homeostatic levels (Cao et al., 2025). This physiological restoration of endogenous HIF-1α activity establishes a bidirectional synergistic effect with EV-induced VEGF secretion (Yuan et al., 2021), cooperatively accelerating the angiogenic cascade in the locally ischemic wound bed.

#### 3.1.2 Umbilical cord-derived mesenchymal stem cell-derived extracellular vesicles (UCMSC-EVs)

Primarily, compelling evidence establishes the central protective role of UCMSC-EVs in oxidative stress-induced skin damage models. Utilizing H_2_O_2_-challenged keratinocytes and UV-irradiated mouse models, demonstrated that UCMSC-EVs alleviate damage across multiple dimensions via adaptive regulation of the NRF2 defense system. They mitigate intracellular ROS accumulation and DNA damage (8-OHdG) while restoring mitochondrial function. Simultaneously, they elevate the activities of antioxidant enzymes (SOD, GSH-PX) and rectify UV-induced anomalous collagen deposition and elevated pro-inflammatory cytokines (TNF-α, IL-1β, IL-6), thereby substantially improving histopathological outcomes Wang et al. (2020a). Mechanistically, this protection is achieved as UCMSC-EVs suppress the oxidative stress-induced aberrant nuclear translocation of NRF2 and the subsequent overactivation of its downstream target genes (e.g., HO-1, NQO1).

Further investigations have elucidated the specific molecular axis through which they combat photoaging. In UVB-irradiated HaCaT cells and murine photodamage models, UCMSC-EVs have been proven to attenuate epidermal thickening, preserve collagen integrity, and alleviate both oxidative stress and inflammation (Liu et al., 2021). The pivotal mechanism driving this effect is the delivery of miR-200a-3p, which establishes the “miR-200a-3p/Keap1/Nrf2″ regulatory axis. By targeting the 3′UTR of Keap1, miR-200a-3p liberates Nrf2 from inhibition, facilitating its nuclear translocation and the subsequent expression of downstream antioxidant genes (SOD2, CAT, NQO1). Concurrently, these EVs synergistically suppress the activation of the NF-κB pathway, thereby downregulating associated pro-inflammatory cytokines and inflammatory enzymes (iNOS, COX-2) to achieve dual antioxidant and anti-inflammatory protection (Gui et al., 2025).

Moreover, hypoxic preconditioning has emerged as an effective approach to enhance the biological activity of UCMSC-EVs. A hypoxic environment induces UC-MSCs to secrete EVs enriched with miR-125 b. These specialized EVs mitigate endothelial cell apoptosis and promote their proliferation and migration by suppressing the target gene TP53INP1, thereby accelerating angiogenesis and cutaneous wound healing in mice (Zhang et al., 2021b). Additional studies across diverse injury models further corroborate the regenerative efficacy of hypoxically preconditioned UCMSC-EVs, attributing these mechanisms to the delivery of specific antioxidant/anti-inflammatory molecules or the activation of pathways such as Wnt/β-catenin (Angelina et al., 2025; Zhang et al., 2015).

#### 3.1.3 Adipose-derived stem cell-derived extracellular vesicles (ADSC-EVs)

The pleiotropic protective effects of ADSC-EVs are well-documented (Zha et al., 2023; Xiong et al., 2020; Liu et al., 2025). In radiation-induced skin injury (RISI) models, ADSC-EVs efficiently scavenge ROS, suppress ROS-mediated apoptosis and pyroptosis, and downregulate the expression of associated executioner proteins (e.g., BAX, Cleaved-Caspase-3, GSDMD-NT, and Caspase-1-P20) (Liu et al., 2025). Concurrently, they mitigate the inflammatory response by reducing the levels of pro-inflammatory cytokines such as IL-1β and IL-6, while actively driving macrophage polarization toward the anti-inflammatory M2 phenotype (CD206^+^) (Liu et al., 2025).

Crucially, the inhibition of ferroptosis constitutes a hallmark mechanism by which hypoxia-preconditioned ADSC-EVs (hypADSC-EVs) exert their protective effects, a process profoundly dependent on their functional RNA cargo. Specifically, the circular RNA circ-Ash1, delivered by hypADSC-EVs, functions as an endogenous miRNA sponge. By sequestering miR-700–5p, circ-Ash1 upregulates the expression of glutathione peroxidase 4 (GPX4), thereby decisively inhibiting lipid peroxidation and executing ferroptosis (Zha et al., 2023). This circ-Ash1/miR-700–5p/GPX4 regulatory axis has been validated as a core pathway in UVB damage models (Zha et al., 2023). Furthermore, high-throughput sequencing suggests that GLRX5, also delivered by hypADSC-EVs, acts synergistically in ROS scavenging and ferroptosis suppression (Liu et al., 2024). These mechanisms are relevant to the oxidative stress injuries precipitated by high-altitude environments.

In terms of promoting tissue regeneration, hypADSC-EVs exhibit dual capacities in anti-aging and collagen remodeling. Studies confirm that they significantly reduce UV-induced senescence-associated β-galactosidase (SA-β-gal) activity alongside p16 and p21 expression, while simultaneously increasing dermal collagen density and downregulating MMP expression in mice (Huynh et al., 2025). Additionally, ADSC-EVs optimize the ratio of ECM components (e.g., elevating type III/I collagen and TGF-β3/TGF-β1 ratios) and inhibit pathological scar formation by activating pathways such as ERK/MAPK (Wang et al., 2017).

### 3.2 Therapeutic potential of other emerging stem cell-derived extracellular vesicles for high-altitude cutaneous damage

Beyond mesenchymal stem cells, EVs derived from induced pluripotent stem cells (iPSCs), epidermal stem cells (EpSCs), hair follicle stem cells (HFSCs), and endothelial progenitor cells (EPCs) have demonstrated substantial regenerative potential in UV- and hypoxia-induced skin injury models. Their fundamental mechanisms revolve around orchestrating critical cellular processes—such as proliferation, collagen metabolism, inflammation, and apoptosis—that meticulously align with the pathological hallmarks of high-altitude damage (Oh et al., 2018; Cui et al., 2024; Liang et al., 2025; Meng and Guo, 2023; Kobayashi et al., 2018).

iPSC-derived extracellular vesicles (iPSC-EVs) possess the unique advantages of unlimited *in vitro* expandability and low immunogenicity, conferring distinct value in cutaneous regeneration (Liang et al., 2025; Meng and Guo, 2023). Empirical evidence confirms that iPSC-EVs promote dermal fibroblast proliferation and migration, upregulate type I collagen synthesis, and concomitantly suppress MMP-1 and MMP-3 expression, thereby effectively reversing UVB-induced cellular senescence (Oh et al., 2018). Furthermore, in diabetic wound models, they accelerate closure and robustly stimulate angiogenesis (Kobayashi et al., 2018); this angiogenic capacity is paramount for ameliorating tissue hypoperfusion precipitated by high-altitude hypoxia (Lin et al., 2025). Mechanistically, iPSC-EVs deliver specific miRNAs (e.g., miR-16–5p, miR-762) to selectively target and modulate signaling cascades, including the p38/MAPK and integrin β1 pathways. This synergistic regulation enhances keratinocyte migration and neovascularization (Yan et al., 2020; Bo et al., 2022), providing a robust strategic rationale for surmounting regenerative impediments in high-altitude environments.

Accumulating evidence highlights the efficacy of EpSC-derived small extracellular vesicles (EpSC-EVs). In vivo cutaneous defect models, these vesicles accelerate wound closure and facilitate the *de novo* regeneration of skin appendages and microvasculature (Zhang et al., 2020). Crucially, EpSC-EVs attenuate endothelial cell injury, restrain excessive autophagic flux, and promote angiogenesis via an intricate signaling network (the miR-200b-3p/SYDE1/RAS/ERK axis) (Xu et al., 2024), providing compelling indirect support for their therapeutic utility in hypoxic microenvironments. Recent investigations further elucidate that EpSC-EVs exert pleiotropic anti-fibrotic, anti-inflammatory, and pro-regenerative effects by delivering miR-203a-3p to suppress the PI3K/AKT/mTOR pathway, or by activating PKN1-cyclin signaling (Zhao et al., 2025; Kang et al., 2024). Nonetheless, their direct mechanisms of action in specific high-altitude injury models warrant further empirical validation.

HFSC-derived small extracellular vesicles (HFSC-EVs) exhibit therapeutic efficacy in UVB-induced photoaging models. By delivering miR-125b-5p, HFSC-EVs activate the TGF-β1/Smad axis, subsequently augmenting collagen synthesis (COL-1, COL-3) and suppressing MMP-1, which ultimately translates to enhanced dermal collagen deposition and wrinkle attenuation *in vivo* (Cui et al., 2024). Furthermore, their documented capacity to suppress pyroptosis and promote tissue repair via the lncRNA H19/NLRP3 axis in diabetic wound models (Yang et al., 2023) highlights their potential therapeutic applicability against the coupled UV and hypoxic stressors of high-altitude environments.

The regenerative paradigm of EPC-derived small extracellular vesicles (EPC-EVs) is fundamentally anchored in their capacity to orchestrate neovascularization. Although direct investigations remain scarce, studies on related endothelial cell-derived EVs confirm their ability to heighten fibroblast metabolic activity and collagen synthesis (Ellistasari et al., 2022). Enriched with pro-angiogenic factors such as VEGF and (fibroblast growth factor 2) FGF-2, these vesicles predominantly drive angiogenesis via the activation of the RAF/ERK pathway, while concurrently mitigating oxidative stress through the suppression of the MAPK/NF-κB cascade (Zhang et al., 2016; Jia et al., 2019; Wu et al., 2018). Their proven efficacy in ameliorating angiogenesis impairments via the AGE-RAGE pathway in diabetic models (Xu et al., 2020) provides a conceptual analog for high-altitude skin therapy, where restoring vascular integrity is paramount. Furthermore, EPC-EVs facilitate collagen maturation and minimize scar formation, exhibiting synergistic functionality with fibroblast-derived EVs (Zhang et al., 2016; Oh et al., 2021).

## 4 Advanced Nano-delivery strategies for high-altitude extreme environments

Despite their therapeutic promise for high-altitude skin injury, the clinical translation of SC-EVs remains constrained by two major barriers: unfavorable pharmacokinetics and limited transdermal delivery. As nanoscale biological vesicles, native EVs are readily diluted within interstitial fluids and rapidly cleared by the mononuclear phagocyte system, resulting in a short local tissue retention time (Parada et al., 2021). More importantly, the dense stratum corneum severely restricts their penetration into deeper dermal layers, leading to inadequate delivery efficiency and suboptimal biodistribution within target tissues (Bos and Meinardi, 2000; Yang et al., 2021a).

These challenges are further exacerbated under the harsh high-altitude microenvironment, where intense ultraviolet radiation, extreme dryness, and low temperatures compromise the structural integrity and physicochemical stability of the EV lipid bilayer. Accordingly, engineered nanodelivery platforms integrated with advanced penetration-enhancement strategies have emerged as a critical approach to improve the stability, retention, and transdermal transport of SC-EVs(Table 2).

**TABLE 2:** Optimization strategies of nanoparticle delivery systems for high-altitude extreme environments.

Strategy classification	Representative materials/Technologies	Plateau-related limitations	Release kinetics	Transdermal and deep-tissue penetration	Biodegradability	Clinical and scalable feasibility	References
Dynamic and Responsive Hydrogel Systems	PVA/gelatin composite hydrogels PLGA-PEG-PLGA GelMA/PEGDA microneedles HA-A/HA-SH	Susceptible to dilution by wound exudates; limited penetration across the dense stratum corneum	Spatiotemporally controlled and sustained release	Limited passive transdermal penetration; microneedle integration enhances dermal-targeted delivery	Enzyme- or hydrolysis-responsive degradation; tunable degradation kinetics	Skin-matched mechanical properties; injectable or thermoresponsive gelation; minimally invasive delivery with reduced pain and material loss	Han et al. (2024b), Wei et al. (2025), Yuan et al. (2022), Yu et al. (2025), Yue et al. (2015), Wang et al. (2019a), Zhu et al. (2022), Kwak et al. (2022), Wang et al. (2019b), Fan et al. (2025), Geng et al. (2022)
EV-Liposome Biomimetic Hybrid Systems	Exosome-liposome fusions (PEG/cholesterol modification) Exosome-coated oxygen nanobubbles Hyaluronic acid-liposomes	Lipid bilayer integrity vulnerable to low-temperature stress	Sustained functional cargo release	Enhanced cellular uptake through lipid composition modulation	Biomimetic lipid bilayer structure with favorable biocompatibility	Preparation dependent on freeze–thaw cycling or sonication; additional lyophilization protection may be required during plateau transport and storage	Han et al. (2024b), Tenchov et al. (2022), Sato et al. (2016), Xie et al. (2025), Koçak et al. (2023), Yang et al. (2025), Lin et al. (2018)
Polymer-Based and Core-Shell Hybrid Systems	Lipid-polymer hybrid nanoparticles (polymer core-lipid shell) Cell membrane-camouflaged PLGA-PEI	Structurally complex formulations; microstructure stability susceptible to transport-associated vibration	Long-term sustained release; H2O2-responsive controlled release	Tunable tissue distribution through regulation of nanoparticle size and surface zeta potential	Gradual biodegradation of polymeric backbones such as PLGA, PEG, and chitosan	Suitable for scalable manufacturing; high encapsulation efficiency and formulation uniformity	Wang et al. (2019b), Wei et al. (2023), Peng et al. (2024), Rabbani et al. (2017), Yang et al. (2021b), Lan et al. (2020)

### 4.1 Optimization of hydrogel-based Nano-delivery systems

Owing to their exceptional biocompatibility, structural mimicry of the ECM, and capacity to maintain a moist microenvironment, hydrogels serve as ideal vehicles for the sustained release of EVs (Yang et al., 2024). The core of their optimal design hinges on modulating cross-linking density and introducing stimuli-responsive and self-healing properties to accommodate the extreme high-altitude environment and complex regenerative demands. This section systematically elucidates the optimization trajectories of hydrogel systems across three dimensions: system design, payload optimization, and preclinical validation.

In terms of system design, functionalized hydrogels adapt to the hostile injury microenvironment through dynamic responsiveness and functional synergy. For instance, a PVA/gelatin composite hydrogel, cross-linked by dynamic borate ester bonds, exhibits distinct shear-thinning and self-healing characteristics. This design averts secondary mechanical trauma, while its intrinsic components mitigate oxidative stress and promote hemostasis (Han et al., 2024b). When subjected to ultrasonic self-assembly with ADSC-Exo-modified oxygen nanobubbles (EBO), the resulting core-shell structures (diameter ∼192 nm, zeta potential −23.30 mV) enhance cellular delivery efficiency and sustain oxygen release for 40 h, effectively ameliorating localized hypoxia and oxidative stress (Han et al., 2024b). Thermoresponsive (e.g., PLGA-PEG-PLGA) and photocrosslinkable (e.g., GelMA/PEGDA) hydrogels have emerged as effective platforms for the controlled release of extracellular vesicles (EVs). Upon exposure to physiological temperature, thermoresponsive hydrogels undergo a sol-gel transition, forming porous networks that retain and gradually release EVs, thereby supporting cell proliferation and angiogenesis (Wei et al., 2025). Photocrosslinkable systems are often integrated with microneedle arrays using UV-assisted curing approaches. These arrays typically exhibit a conical geometry, with heights of approximately 600 μm and base diameters near 300 μm. *In vitro* studies have shown sustained cumulative EV release exceeding 80% over 10 days, while *in vivo* fluorescence tracking demonstrated prolonged local retention for up to 8 days. Compared with conventional injection or topical spraying, microneedle-assisted delivery improves EV penetration into deeper tissue while reducing procedural discomfort and formulation loss (Yuan et al., 2022). Additionally, injectable hydrogels based on thiol-ene click chemistry (e.g., HA-A/HA-SH) undergo rapid gelation at room temperature. Their mechanical properties (storage modulus, G' ∼1.6 kPa) are compatible with native skin, conferring a biomechanically matched microenvironment for tissue regeneration (Yu et al., 2025).

Regarding payload optimization and functional expansion, chemical cross-linking (e.g., GelMA) or stimuli-responsive architectures can drastically elevate loading efficiency and facilitate on-demand release (Yue et al., 2015; Wang et al., 2019a). For example, a lysozyme-sensitive GelMA/SFMA hydrogel exhibits accelerated degradation within the inflammatory microenvironment, synergistically releasing EVs and anti-inflammatory therapeutics to drive the polarization of macrophages toward the reparative M2 phenotype (Zhu et al., 2022). A hydrolysis-sensitive 8 P-TS PEG hydrogel features an adjustable degradation profile ranging from 6 to 27 days. This prolongs the localized retention of therapeutic EVs concentrations, thereby promoting the regeneration of skin appendages and adipose tissue (Kwak et al., 2022), providing a valuable reference for long-term regenerative strategies at high altitudes. Further integration of antimicrobial peptides or pH-responsive elements enables the construction of multifunctional platforms that seamlessly couple infection control with tissue repair (Wang et al., 2019b).

Accumulating preclinical evidence substantiates the regenerative efficacy of these systems, with their core mechanisms meticulously aligning with the therapeutic imperatives of high-altitude skin injuries. In a rat full-thickness cutaneous wound model, the PVA/GA system expedited wound closure, attenuated inflammation (evidenced by reduced IL-6 levels and a modulated M1/M2 macrophage ratio), and stimulated both angiogenesis and collagen deposition (Han et al., 2024b). The PLGA-PEG-PLGA hydrogel encapsulating SC-EVs achieved a 98.6% wound closure rate by day 14 post-surgery. Mechanistically, this was driven by early-stage promotion of angiogenesis (upregulated CD31), accelerated tissue remodeling (robust α-SMA expression and enhanced collagen deposition), and the suppression of hyperinflammation (downregulated TNF-α) (Wei et al., 2025). Even in challenging diabetic or infected wound models, various hydrogel platforms (such as SISMA/H-exo, GelMA/PEGDA microneedles, CEC-DCMC, and FHE) demonstrated improved performance. They effectively prolonged EVs retention, modulated the immune microenvironment (inducing M1-to-M2 polarization), and activated pro-regenerative pathways (e.g., VEGF/PI3K/AKT) to synergistically orchestrate neovascularization, re-epithelialization, and collagen remodeling (Yuan et al., 2022; Yu et al., 2025; Wang et al., 2019b; Fan et al., 2025; Geng et al., 2022).

### 4.2 Optimization of liposomal and polymeric Nano-delivery systems

Owing to their lipid bilayer architecture, which is analogous to that of EVs, liposomes offer distinct advantages in biocompatibility and payload delivery, rendering them ideal vehicles for the transdermal delivery of EVs. To combat the cutaneous damage provoked by the high-altitude environment, optimization strategies predominantly focus on tailoring membrane composition, controlling particle size, and hybridizing with EVs. These approaches aim to augment both environmental adaptability and regenerative efficacy. For instance, the incorporation of cholesterol or sphingomyelin can significantly enhance the thermodynamic stability and biocompatibility of the liposomal membrane (Tenchov et al., 2022). Concurrently, EV-liposome hybridization strategies synergistically combine the intrinsic advantages of both entities, drastically elevating delivery efficiency and bioactivity.

Specifically, freeze-thaw cycling has been successfully employed to fabricate EVs-liposome hybrids with high membrane fusion efficiency. Cellular uptake of these hybrids can be further optimized by modulating the lipid composition (e.g., via PEGylation) (Sato et al., 2016). To augment the hypoxia tolerance of the delivery vehicle, an advanced EVs-modified system can be constructed by conjugating glycan sulfate with bovine serum albumin to form nanoparticles (diameter 122.50 nm), generating oxygen nanobubbles via ultrasound-mediated oxygenation, and finally encapsulating them with an EVs coating at a 1:2 ratio via sonication (Han et al., 2024b). Furthermore, hybrid nanoparticles (diameter ∼190 nm)—constructed by fusing neural stem cell-derived EVs with drug-loaded liposomes—not only preserve the targeting specificity of EVs and enhance loading efficiency but also exert profound cytoprotective effects in ischemic and hypoxic models. Mechanistically, they achieve this by suppressing pro-inflammatory cytokines (such as TNF-α and IL-6) and activating the AKT/Nrf2/HO-1 antioxidant pathway (Xie et al., 2025). Similarly, a hybrid system integrating umbilical cord blood EVs with lecithin liposomes significantly promotes the proliferation and migration of dermal fibroblasts and keratinocytes while alleviating oxidative stress damage, primarily by upregulating regeneration-associated factors such as COL1A1 and VEGF (Koçak et al., 2023). In laser-induced photodamage models, EVs encapsulated within hyaluronic acid-liposome hybrids accelerate collagen regeneration and wound closure by downregulating IL-6 and TNF-α levels (Yang et al., 2025). This hybridization paradigm has even been extended to gene delivery, providing a technological avenue for utilizing the CRISPR/Cas9 system to orchestrate hypoxia-adaptation targets within mesenchymal stem cells (Lin et al., 2018).

In contrast to liposomes, polymeric nanoparticles—represented by PLGA, PEG, and chitosan—encapsulate EVs predominantly via electrostatic interactions or self-assembly (Wei et al., 2023). Continuous technological optimization has further augmented the translational value of these nanocarriers. For example, a smart hydrogel system constructed from diverse polymers enables the H_2_O_2_-responsive, controlled release of EVs (achieving a 90% release rate over 28 days). In diabetic wound models, this system delivers specific miRNAs to synergistically suppress inflammation and stimulate angiogenesis (Wang et al., 2019b). Another pivotal study utilized adipose-derived stem cell membrane-camouflaged PLGA-PEI nanocarriers to deliver miR-21. By targeting the PTEN gene and subsequently activating the AKT pathway, this system finely tunes the inflammatory response and promotes collagen remodeling, thereby accelerating wound healing (Peng et al., 2024).

To synergize the excellent biocompatibility of liposomes with the superior structural stability of polymers, lipid-polymer hybrid nanoparticles (LPNs)—characteristically featuring a “polymer core-lipid shell” architecture—have been developed. The fabrication methods for these systems (such as emulsion-solvent evaporation and nanoprecipitation) readily facilitate scalable production. Their performance is optimized through the precise manipulation of particle size (typically concentrated within 50–300 nm), Zeta potential (approximately −10 to −25 mV), and surface modification (e.g., PEGylation). Notably, their encapsulation efficiency frequently exceeds 80%, accompanied by exceptional formulation homogeneity (polydispersity index, PDI <0.2) (Rabbani et al., 2017; Yang et al., 2021b; Lan et al., 2020). Functionally, these hybrid systems achieve the progressive, sustained release of active therapeutic payloads within wound models. They effectively mitigate the inflammatory cascade by activating antioxidant pathways (e.g., Nrf2) and downregulating the expression of pro-inflammatory cytokines (IL-6, TNF-α). Concurrently, they upregulate angiogenic markers (CD31, α-SMA) to propel robust collagen deposition and re-epithelialization, thereby providing multi-dimensional support for cutaneous regeneration (Rabbani et al., 2017; Yang et al., 2021b; Lan et al., 2020).

Furthermore, the direct engineering of nanosystems via EVs membrane reconstitution represents a crucial trajectory. For instance, sonication-mediated fabrication of oxygenated EVs-hemoglobin nanoparticles (diameter 100–130 nm) enables sustained oxygen delivery (≥10 h). By downregulating the expression of HIF-1α and inflammatory cytokines (IL-6, IL-8), this system effectively drives the proliferation and migration of epithelial cells, ultimately expediting wound closure under hypoxic conditions (Han et al., 2025). This distinctive attribute endows it with substantial translational potential for repairing oxidative stress-induced skin injuries.

## 5 Practical barriers to clinical translation: limitations in the evidence framework and biological risks under extreme conditions

The clinical translation of SC-EVs combined with nanodelivery systems for high-altitude skin injury remains constrained by multiple translational barriers. A primary limitation lies in the mismatch between current preclinical models and the complex pathological landscape of high-altitude environments, together with substantial methodological heterogeneity across studies. Most existing therapeutic evidence is derived from normoxic lowland models or single-stressor injury systems, which fail to adequately recapitulate the multidimensional and coupled pathological features of high-altitude skin injury. In addition, the lack of standardization in EV isolation protocols, characterization methods, and dosing strategies further compromises cross-study comparability. As such, directly extrapolating reparative effects observed in simplified experimental settings to high-altitude conditions remains insufficiently rigorous. Future studies should prioritize the development of high-fidelity *in vivo* models that better simulate composite high-altitude stressors to establish a more robust preclinical evidence framework.

From a biosafety perspective, the multitarget regulatory properties of EVs may also carry a risk of maladaptive responses under extreme microenvironmental conditions. One major concern involves dysregulated vascular and matrix remodeling. In the setting of endothelial injury and impaired microcirculatory compensation at high altitudes, excessive pro-angiogenic signaling from EV-based therapies may promote the formation of immature and hyperpermeable microvessels, thereby aggravating tissue edema. Similarly, under sustained hypoxia-associated HIF-1α activation, excessive stimulation of the TGF-β/Smad pathway may disturb extracellular matrix homeostasis and increase the risk of pathological fibrosis.

Another unresolved concern relates to tumor-promoting potential. Because chronic ultraviolet exposure at high altitudes is itself a potent driver of DNA damage and mutagenesis, the intrinsic pro-survival and pro-proliferative properties of EVs may theoretically facilitate the expansion or persistence of pre-existing premalignant cells, although direct evidence remains limited.

Beyond these mechanistic concerns, the systemic exposure profile and immunological consequences of EV-based therapies also remain poorly defined. Extensive barrier disruption in high-altitude wounds may increase the risk of unintended systemic entry of locally administered EVs through damaged vasculature. However, their long-term biodistribution, clearance kinetics, and potential tissue accumulation following uptake by the mononuclear phagocyte system have not yet been systematically evaluated. In parallel, although EVs may suppress excessive inflammatory cascades in high-altitude wounds, prolonged or unintended immunomodulation could impair local T-cell or NK-cell surveillance, potentially increasing susceptibility to opportunistic infections.

## 6 Translational pathways, regulatory considerations, and precision intervention strategies for cell-free therapies in high-altitude skin injury

### 6.1 Regulatory classification and population-oriented precision intervention strategies

Advancing the clinical translation of extracellular vesicle (EV)-based therapies for high-altitude skin injury first requires clear definition of their regulatory classification and compliance framework. According to current guidance from the International Society for Extracellular Vesicles, as well as regulatory perspectives from the European Medicines Agency and the U.S. Food and Drug Administration, native stem cell-derived EVs without genetic modification are generally considered biological products because they originate from endogenous intercellular communication processes. In many contexts, they are not automatically categorized as high-risk advanced therapy medicinal products (ATMPs) or gene therapy products, which may provide a more feasible pathway for early-stage clinical translation.

Within this regulatory framework, therapeutic strategies for high-altitude skin injury should account for substantial interindividual heterogeneity in genetic background, environmental adaptation, and pathological susceptibility. Indigenous high-altitude populations have developed adaptive mechanisms related to hypoxia tolerance and pigmentation regulation through long-term evolutionary selection (Yang et al., 2022, Schrempf et al., 2016), whereas short-term exposed individuals lacking such adaptations are more susceptible to excessive oxidative stress and inflammatory amplification under combined environmental stressors. In addition, age-associated decline in dermal fibroblast activity (Wang et al., 2020b; Zhang et al., 2024b), together with the limited intrinsic photoprotection of individuals with Fitzpatrick skin types I–III, may further increase vulnerability to local tissue damage (Del Bino et al., 2018; Brar et al., 2025; Battie and Verschoore, 2012).

Accordingly, EV-based therapies should adopt stratified intervention strategies tailored to both patient characteristics and disease stage, with treatment selection guided by the intended mode of action (MoA). For acute exposure or early-stage injury, the primary therapeutic objective is to suppress apoptosis and attenuate excessive inflammatory activation through modulation of macrophage polarization. In this setting, injectable hydrogels may serve as localized delivery platforms for EVs enriched in anti-inflammatory or anti-ferroptotic factors, thereby improving retention within deep tissue compartments. By contrast, in chronic high-altitude skin injury characterized by impaired tissue remodeling, the therapeutic focus shifts toward promoting microvascular regeneration and restoring ECM homeostasis. Under these conditions, advanced transdermal delivery systems such as microneedle arrays may facilitate sustained and quantitatively controlled delivery of EVs and pro-angiogenic cargos across the resistant stratum corneum barrier.

The successful implementation of this precision-therapy framework will ultimately depend on high-quality prospective randomized controlled trials following a stepwise translational strategy. Future clinical studies should establish independent cohorts for acute and chronic exposure conditions and integrate multimodal noninvasive assessment approaches rather than relying solely on subjective clinical scoring systems. Dynamic evaluation of transepidermal water loss (TEWL), microvascular Doppler perfusion reserve, and the subepidermal low-echogenic band (SLEB) detected by high-frequency ultrasound may provide important macroscopic indicators of barrier integrity and dermal remodeling. These parameters should be further combined with molecular profiling of lipid peroxidation and DNA damage biomarkers to establish a multidimensional evidence framework linking mechanistic regulation with clinical phenotypes, thereby supporting the standardized clinical development of EV-based therapies for high-altitude skin injury.

### 6.2 Scalable GMP-Compliant manufacturing and mechanism-oriented quality control frameworks

A major obstacle preventing the large-scale clinical translation of EVs is the establishment of robust chemistry, manufacturing, and controls (CMC) systems together with standardized good manufacturing practice (GMP) workflows. During upstream production, stringent control of donor-cell traceability and passage number is essential, while the use of chemically defined serum-free media or humanized alternatives is increasingly regarded as a prerequisite for industrial-scale manufacturing.

At the purification stage, conventional ultracentrifugation remains difficult to adapt for clinical-scale production because of its limited scalability, risk of vesicle aggregation, and frequent co-isolation of contaminating proteins. Future manufacturing strategies will likely shift toward low-shear integrated purification platforms, including tangential flow filtration (TFF) combined with size-exclusion chromatography (SEC) or field-flow fractionation (FFF), in order to improve production yield while preserving EV structural integrity and biological activity.

At the downstream quality-control (QC) level, current release criteria based primarily on static physicochemical characterization—such as nanoparticle tracking analysis (NTA) or transmission electron microscopy (TEM)—remain insufficient to predict therapeutic performance under the complex pathological conditions associated with high-altitude skin injury. QC systems should therefore follow the Minimal Information for Studies of Extracellular Vesicles (MISEV) guidelines, including enrichment analysis of canonical membrane markers (e.g., CD63, CD9, and CD81), exclusion of intracellular contaminants such as calnexin, and comprehensive testing for *mycoplasma*, endotoxins, and microbial sterility. More importantly, customized biological potency assays tailored to the pathological features of high-altitude skin injury are needed to minimize efficacy drift during scale-up manufacturing and to ensure batch-to-batch consistency in clinical safety and therapeutic efficacy.

### 6.3 Pharmaceutical stability and intelligent cold-chain logistics for extreme environments

The dramatic climatic fluctuations and prolonged transportation conditions associated with high-altitude regions present substantial stability challenges for EV formulations, particularly because the lipid bilayer membrane and surface-associated proteins are sensitive to physical stress. Future strategies will likely depend on optimized lyoprotectant formulations, such as trehalose, together with secondary polymer encapsulation approaches to improve stability during dehydration and long-term storage (Qazi et al., 2023; Li et al., 2021).

**FIGURE 3:**
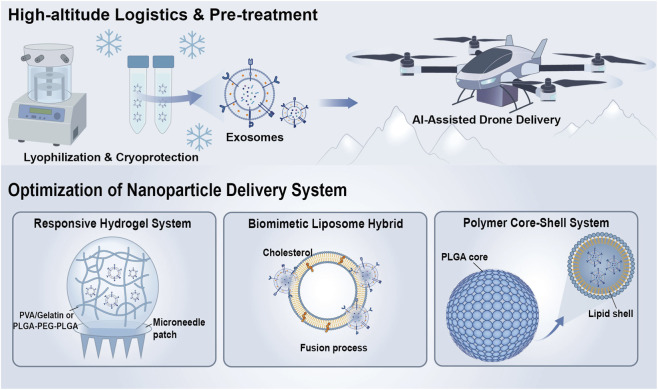
Optimization of Extracellular Vesicles Nanoparticle Delivery Systems and Intelligent Logistics Framework Adapted for High-Altitude Environments. (All panels are original illustrations created by the authors using Adobe Illustrator).

Building upon these pharmaceutical stabilization strategies, further integration of artificial intelligence (AI), the Internet of Things (IoT), and unmanned aerial vehicle (UAV)-based logistics systems may improve transportation efficiency and environmental monitoring in remote high-altitude settings(Figure 3). Emerging evidence from high-altitude delivery studies suggests that the combination of environmentally resilient nanotherapeutics with intelligent transport networks may help overcome geographical and climatic barriers to medical supply distribution, thereby improving the feasibility of off-the-shelf EV-based therapeutics for high-altitude applications [27].

## 7 Conclusion

In summary, SC-EVs demonstrate considerable therapeutic potential for mitigating complex skin injury under high-altitude conditions. However, substantial barriers still limit their translation from bench to bedside. Future progress will require the development of high-fidelity composite disease models to strengthen the preclinical evidence base, together with precision intervention strategies that account for population heterogeneity and disease stage.

From both manufacturing and regulatory perspectives, the establishment of GMP-compliant large-scale production pipelines and mechanism-oriented biological potency evaluation systems will be critical for ensuring product consistency, environmental adaptability, and regulatory compliance. At present, the convergence of stem cell biology and advanced nanodelivery technologies has already provided a practical engineering framework for overcoming the transdermal barrier associated with high-altitude skin injury. Looking forward, the continued integration of intelligent storage and transport systems may further improve the clinical accessibility of EV-based therapies in geographically remote and environmentally extreme regions, thereby facilitating the development of precision regenerative strategies for skin protection and repair at high altitudes.
